# Water-Soluble and Freezable Aluminum Salt Vaccine Adjuvant

**DOI:** 10.3390/vaccines12060681

**Published:** 2024-06-19

**Authors:** Erwin G. Abucayon, Ilya Belikow-Crovetto, Elizabeth Hussin, Jiae Kim, Gary R. Matyas, Mangala Rao, Carl R. Alving

**Affiliations:** 1 Henry M. Jackson Foundation for the Advancement of Military Medicine, 6720A Rockledge Drive, Bethesda, MD 20817, USA; eabucayon@hivresearch.org (E.G.A.); jakim@hivresearch.org (J.K.); 2 U.S. Military HIV Research Program, Center for Infectious Diseases Research, Walter Reed Army Institute of Research, 503 Robert Grant Avenue, Silver Spring, MD 20910, USA; ibelikow@hivresearch.org (I.B.-C.); gmatyas@hivresearch.org (G.R.M.); mrao@hivresearch.org (M.R.); 3 Oak Ridge Institute for Science and Education, Oak Ridge, TN 37831, USA

**Keywords:** vaccine adjuvant, cold chain, aluminum salt, aluminum chloride, aluminum triacetate, aluminum sulfate

## Abstract

Particulate aluminum salts have long occupied a central place worldwide as inexpensive immunostimulatory adjuvants that enable induction of protective immunity for vaccines. Despite their huge benefits and safety, the particulate structures of aluminum salts require transportation and storage at temperatures between 2 °C and 8 °C, and they all have exquisite sensitivity to damage caused by freezing. Here, we propose to solve the critical freezing vulnerability of particulate aluminum salt adjuvants by introducing soluble aluminum salts as adjuvants. The solubility properties of fresh and frozen aluminum chloride and aluminum triacetate, each buffered optimally with sodium acetate, were demonstrated with visual observations and with UV–vis scattering analyses. Two proteins, A244 gp120 and CRM_197_, adjuvanted either with soluble aluminum chloride or soluble aluminum triacetate, each buffered by sodium acetate at pH 6.5–7.4, elicited murine immune responses that were equivalent to those obtained with Alhydrogel^®^, a commercial particulate aluminum hydroxide adjuvant. The discovery of the adjuvanticity of soluble aluminum salts might require the creation of a new adjuvant mechanism for aluminum salts in general. However, soluble aluminum salts might provide a practical substitute for particulate aluminum salts as vaccine adjuvants, thereby avoiding the risk of inactivation of vaccines due to accidental freezing of aluminum salt particles.

## 1. Introduction

In 1926, Glenny and colleagues initiated the field of aluminum salts as vaccine adjuvants when they demonstrated that precipitation of diphtheria toxoid with potassium alum [AlK(SO_4_)_2_·12H_2_O] resulted in an insoluble material, which, upon injection into guinea pigs, resulted in an enhanced immune response to the toxoid [1]. From this simple beginning, the number and impact of vaccines containing aluminum salts have burgeoned dramatically. A 2012 analysis by PATH of worldwide vaccines revealed that, among the total of 235 vaccines identified and studied, 146 were adjuvanted with an aluminum salt [2]. As of 2023, among 96 vaccines that were licensed by the US Food and Drug Administration [3], 30 contained aluminum salt as an adjuvant [4]. The worldwide distribution and resultant benefits of millions of annual doses of aluminum-adjuvanted vaccines for protection against diseases such as diphtheria, tetanus, pertussis, pneumococcal pneumonia, various cancers (induced by human papilloma and hepatitis B viruses), hepatitis A, and hepatitis B have been a miracle of vaccinology and modern public health [5,6].

Although the historical benefits accrued by the use of particulate aluminum salt adjuvants are well documented, the mineralogical and chemical properties of the particles can be quite complex [7]. Alum precipitation of an antigen is now rarely used, having been superseded by adsorption of the antigen to preformed gels of particles of aluminum hydroxide (AH) or aluminum phosphate (AP). Depending on their mineralogical and chemical properties, the multistep preparation of particulate aluminum salt and adsorption of antigen to the surface of the particles is complicated, as reviewed in 2018 [7]. Even the inability to sterile-filter the manufactured vaccine because of the micron-size of the aggregated aluminum salt particles poses problems.

However, the greatest danger to adjuvanted vaccines lies after manufacture when the vaccines are released and required to adhere rigidly to the cold chain refrigeration storage requirement: “Store refrigerated between 2 °C and 8 °C (36 °F and 46 °F). Do not freeze. Discard if the vaccine has been frozen [8]”. This temperature requirement is critical because freezing irreversibly damages the structure of the aluminum particle, thus reducing or eliminating the binding, or changing the presentation, of the adsorbed protein antigen. Numerous studies have demonstrated the extreme danger of freezing, in which the aluminum particle structure is irreversibly damaged or destroyed by even a single event of freezing, resulting in protein antigen desorption, and reduction or loss of adjuvanticity, as described in numerous studies [9,10,11,12,13].

After manufacturing of the aluminum-adjuvanted vaccines is completed, millions of doses are distributed annually through cold chains worldwide. The cold chain includes the transportation and storage of the vaccine; however, a disturbing 2007 meta-analysis review of the published literature and unpublished documents between 1985 and 2006 concluded that “…accidental freezing [of vaccines] is pervasive and occurs across all segments of the cold chain [14]”. A second meta-analysis review 10 years later that excluded data from the 2007 report suggested that about one-third of the vaccines in the cold chain in both developed and developing countries were accidentally exposed to temperatures below the recommended ranges [15].

Keeping in mind the vulnerability of aluminum adjuvant particles and the absolute need to avoid even a single event of freezing, we hypothesized that the worldwide problem of structural disruption of particulate aluminum salt caused by accidental freezing of adjuvanted vaccines might be overcome by utilizing soluble, rather than particulate, aluminum salts as adjuvants. Here, we have discovered that certain soluble aluminum salts do have adjuvant potencies that are equivalent to those induced by a widely used commercial particulate aluminum hydroxide adjuvant (Alhydrogel^®^) for enhancing production of murine antibodies to gp120, which is an envelope protein of HIV-1, and CRM_197_, which is a nontoxic protein variant of diphtheria toxin.

## 2. Materials and Methods

### 2.1. Materials and Reagents

Aluminum chloride hexahydrate (AlCl_3_·6H_2_O) (catalog number A0718), aluminum sulfate octahydrate (Al_2_(SO_4_)_3_·8H_2_O) (catalog number 1017364), and sodium acetate (NaCOOCH_3_) (catalog number S5636) were purchased from Sigma-Aldrich, Inc. (St. Louis, MO, USA). Aluminum triacetate, also known as aluminum acetate (Al(COOCH_3_)_3_) (catalog number NCZ-AE-104/21) was purchased from NanoChemazone (Leduc, AB, Canada).

Antigens A244 gp120 and CRM_197_ were purchased from Immune Technology, Inc. (New York City, NY, USA), and Fina Biosolutions, LLC (Rockville, MD, USA), respectively. Horseradish peroxidase (HRP)-conjugated goat anti-mouse IgG was purchased from Origene (Rockville, MD, USA). HRP-conjugated goat anti-mouse IgG2a and mouse IgG2a antibody were purchased from Southern Biotech. The KPL ABTS Peroxidase 2-component substrate system was purchased from SeraCare (Gaithersburg, MD, USA). Immulon^®^ 96-well U-bottom plates and NUNC Maxisorp^TM^ 96-well flat-bottom plates were purchased from Thermo Fisher Scientific (Waltham, MA, USA). Dulbecco’s phosphate-buffered saline (DPBS) without calcium and magnesium, 10% sodium dodecyl sulfate (SDS), powdered nonfat milk, and 10× phosphate-buffered saline were purchased from Quality Biological, Inc. (Gaithersburg, MD, USA). Tween^®^ 20 was purchased from Millipore Sigma (St. Louis, MO, USA). Powdered nonfat milk was purchased from a local grocery store. Alhydrogel^®^ (aluminum hydroxide gel) was purchased from Brenntag, Inc. (Reading, PA, USA), and Croda, Inc. (Princeton NJ, USA). Sterile filtration was conducted with a polyvinylidene difluoride (PVDF) syringe (VWR, catalog number 76478-992, Bridgeport, NJ, USA). Stock solutions were prepared in ultrapure water purchased from KD Medical (Columbia, MD, USA). pH measurements were taken with a Thermo Scientific Orion VersaStar Pro pH meter equipped with an Orion Ross Microprobe (Waltham, MA, USA).

### 2.2. Preparation of Adjuvants

#### 2.2.1. Aluminum Chloride as an Adjuvant

A fresh stock solution of 40 mg/mL aluminum chloride (4.47 mg Al/mL) was prepared in ultrapure water or DPBS. From this stock solution, 1.125 mL was added to 2.235 mL 5 M sodium acetate buffer or DPBS to obtain a final concentration of 1.5 mg/mL Al at pH ~6.5–7.0 or pH ~2.9–3.5, respectively. The final adjuvant solution was sterile-filtered through a 0.22 μm PVDF syringe filter in a biosafety cabinet (BSC).

#### 2.2.2. Aluminum Triacetate as an Adjuvant

A fresh stock solution of 33.9 mg/mL aluminum triacetate (4.48 mg Al/mL) was prepared by dissolving aluminum triacetate powder in ultrapure water. From this stock solution, 1.125 mL was added to 2.235 mL 5 M sodium acetate buffer to obtain a 1.5 mg/mL final concentration of Al at pH ~6.5–7.0. Similarly, 0.56 mL of the stock solution and 0.56 mL of ultrapure water were added to 2.235 mL of 5 M sodium acetate buffer to obtain a 0.75 mg/mL final concentration of Al. The final adjuvant solutions were sterile-filtered through 0.22 μm PVDF syringe filters in a BSC.

#### 2.2.3. Alhydrogel^®^ (Aluminum Hydroxide Gel (AH)) Adjuvant

An aliquot of 0.182 mL of stock solution (10.3 mg Al/mL) of AH was added to 2.318 mL of 0.1 M sodium acetate buffer (pH ~6.5) or DPBS (pH ~7.4) to obtain a concentration of 0.75 mg/mL Al (pH ~6.6 or ~7.5, respectively). The preparation was performed in a BSC.

### 2.3. Adjustment of Formulation pH

**Figure 1:**
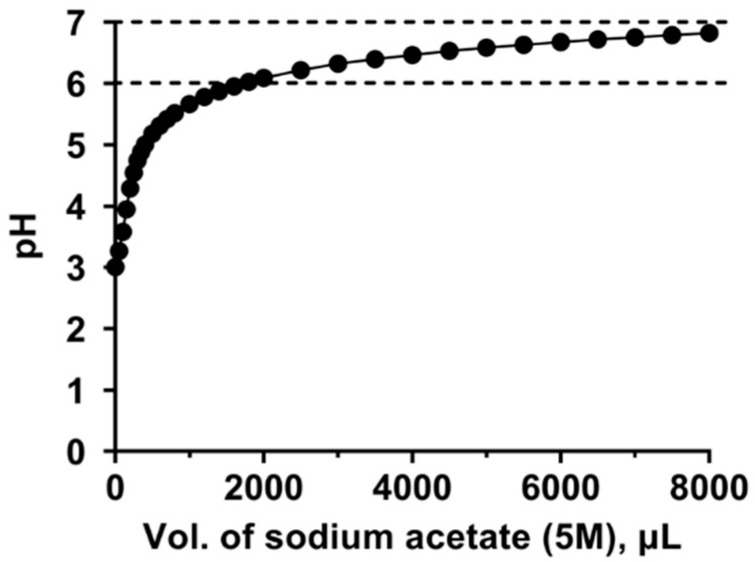
Titration of aluminum chloride, 40 mg/mL (4.47 mg Al/mL) in water, to pH 6.0–7.0 with 5 M sodium acetate buffer (pH = 9.0). Each point represents the mean ± SD of the resulting pH (*n* = 3). The SDs are too small to be seen in the figure.

A solution of 40 mg/mL aluminum chloride (4.47 mg Al/mL) in ultrapure water with a starting pH of ~2.9 was titrated against 5 M sodium acetate (pH ~9.0). An incremental volume of 50, 100, 200, or 500 μL of 5 M sodium acetate was added to the aluminum chloride solution and stirred for ~1 min, and the pH was recorded using a calibrated pH meter. A pH range of 6.0–7.0 was obtained after the addition of a total volume of 2–8 mL of 5 M sodium acetate. The titration curve for the adjustment of the pH of aluminum chloride solution with 5 M of sodium acetate is presented in Figure 1.

### 2.4. Physico-Chemical Characterization of Adjuvant

#### 2.4.1. Visual Characterization

**Figure 2:**
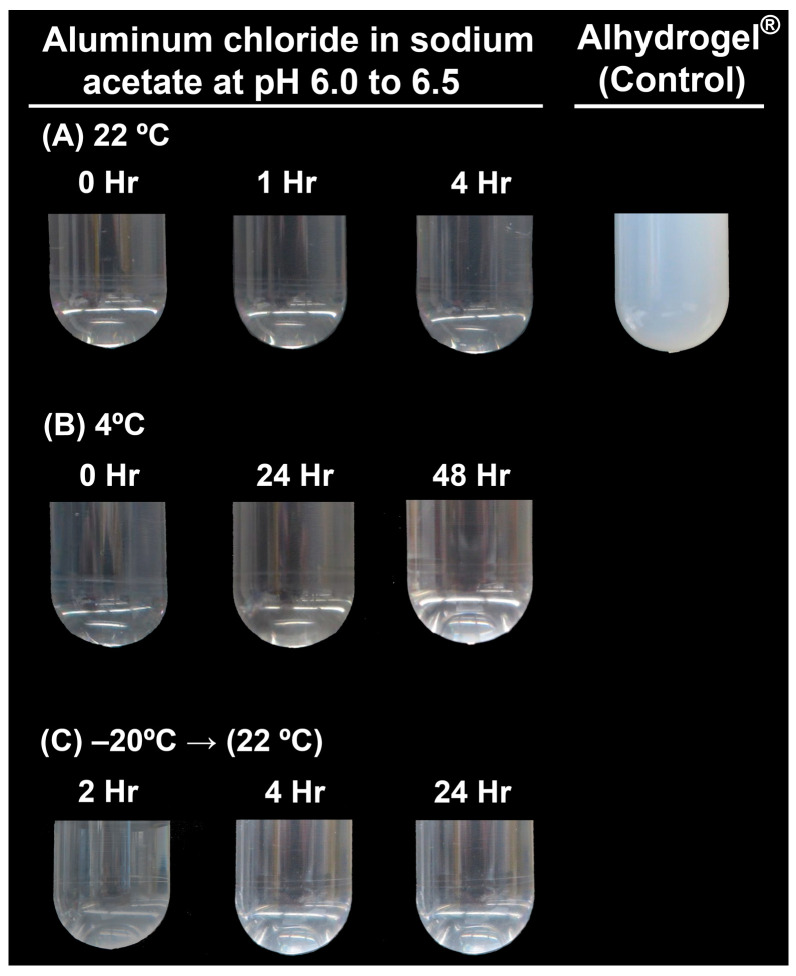
Physical appearance of aluminum chloride adjuvant in sodium acetate buffer, pH = 6–7, under different conditions. Alhydrogel^®^ was used as a reference. Photographs were taken immediately (0 h) or after 1, 2, 4, 24, and 48 h at 22 °C. (**A**) Freshly prepared adjuvant at 22 °C, (**B**) freshly prepared adjuvant stored at 4 °C, and (**C**) frozen adjuvant at −20 °C and thawed at 22 °C.

The changes in the physical appearance of the aluminum chloride adjuvant in 5 M sodium acetate prepared under different storage conditions were investigated as a function of time. Aluminum chloride adjuvant stored at 22 °C was observed at 0, 1, and 4 h; aluminum chloride adjuvant stored at 4 °C was observed at 0, 24, and 48 h; aluminum chloride adjuvant frozen at −20 °C was slowly thawed at 22 °C and observed at 0, 2, 4, and 24 h. Photographs were taken at every time point using a Canon PowerShot G1 X Mark II camera (Ōta, Tokyo, Japan). To minimize the ambient reflective light from the outside surfaces of the tubes in Figure 2, the brightnesses of all the composite images were adjusted together to improve the transparent image clarity.

#### 2.4.2. UV–Visible Scattering Measurement

An amount of 1.125 mL of 40 mg/mL aluminum chloride (4.47 mg Al/mL) or 33.9 mg/mL aluminum triacetate (4.48 mg Al/mL) was added to 2.235 mL of 5 M sodium acetate buffer to obtain a final concentration of 1.5 mg/mL Al for each aluminum solution. A separate suspension of 0.75 mg Al/mL Alhydrogel was prepared by adding 0.15 mL of 10.3 mg Al/mL Alhydrogel to 1.85 mL of 5 M sodium acetate. The 1 mL aliquots of each final solution (1.5 mg/mL Al) were stored at −20 °C overnight. An amount of 200 μL of each remaining solution or suspension was transferred to a 96-well Corning UV-transparent flat-bottom plate. A negative control consisted of 5 M sodium acetate. The absorbance scan from 300 nm to 700 nm was then collected on a BioTek Epoch2 Microplate Reader (Agilent, Santa Clara, CA, USA). On the following day, the frozen solutions were slowly thawed and equilibrated to RT, and their absorbances were measured in a similar manner as described above. The absorbance profiles of each freshly prepared 1.5 mg/mL Al in aluminum chloride and Alhydrogel in DPBS were also measured as described above.

#### 2.4.3. Adsorption Capacity Measurement

The antigen adsorption capacities of the Alhydrogel in PBS and sodium acetate were indirectly determined from the protein antigen measured in the supernatant by BCA assay after centrifugation. The sample was prepared by adding 140 μL of A244 gp120 (1 mg/mL) to 560 μL AH (0.75 mg Al/mL) in 100 mM sodium acetate or DPBS in sterile 1.5 mL microcentrifuge tubes. The formulations were mixed in a roller for 15 min, followed by incubation at 4 °C for 1 h. The samples were then centrifuged at 21,130× *g* for 10 min, and the supernatants were recovered carefully.

The amount of protein antigen in the supernatant was determined by BCA assay as per the manufacturer’s instruction (Pierce^TM^, Rockford, IL, USA, Thermo Scientific). Briefly, a series of BSA calibration standards in the range of 25–1000 mg/mL in DPBS was prepared. A working reagent (WR) was then prepared by adding 50 parts of Pierce BCA Protein Assay Reagent A to 1 part of Pierce BCA Protein Assay Reagent B. We used 25 μL in triplicate of each calibration standard, and the samples were pipetted into the 96-well plate. An amount of 200 μL of WR was then added to each well, and the plate was placed in the shaker for 30 s. The plate was then covered with optical adhesive film and incubated at 37 °C for 30 min. The absorbance was measured at 562 nm. The protein antigen concentrations in the samples were interpolated from the regression analysis of the calibration standards. The percent (%) adsorption was determined from the equation below. The A244 gp120 adsorption capacities for Alhydrogel in DPBS and in sodium acetate were 96.26 ± 0.64% and 100%, respectively.

$$\%\;\mathrm{Adsorption}=(\frac{\left(\left[added\;antigen\right]-[antigen\;in\;the\;supernatant]\right)}{\left[added\;antigen\right]})\times 100$$

### 2.5. Vaccine Formulation

Vaccines were prepared by mixing appropriate amounts of antigen and adjuvant to generate formulations containing 10 μg of HIV-1 A244 gp120 or CRM_197_ and 30, 60, or 90 μg of Al in 50 μL immunizations. Briefly, 140 μL of A244 gp120 (1 mg/mL) or CRM_197_ (1 mg/mL) was mixed with 560 μL aluminum chloride (1.5 mg/mL of Al) or AH (1.5 mg/mL of Al) in 5 M sodium acetate or DPBS in a sterile microfuge tube. The vaccine formulations were further mixed by shaking on a roller for 15 min, followed by incubation at 4 °C for 1 h prior to immunization. The final pH values of all vaccine formulations were in the range of 6.0–7.4.

### 2.6. Immunization of Mice

#### 2.6.1. Ethics Statement

Animal studies were carried out in accordance with the recommendations in the Guide for the Care and Use of Laboratory Animals of the National Institutes of Health. The protocols were approved by the Institutional Animal Care and Use Committee at the Walter Reed Army Institute of Research [Assurance number D16-00596 (A4117-01)]. Blood collection was performed as per WRAIR/NMRC VSP Guidelines on blood collection.

#### 2.6.2. Animal Immunization

Female BALB/c mice (5–6 weeks of age) were obtained from The Jackson Laboratory. Animals were housed in groups and fed standard chow diets. Mice (N = 5 or 10) were immunized intramuscularly in the hind-leg quadriceps. All immunizations contained 10 μg of HIV-1 A244 gp120 or CRM_197_, with different types of aluminum adjuvants (i.e., aluminum chloride in sodium acetate buffer, aluminum chloride in DPBS, aluminum triacetate in 5 M sodium acetate buffer, aluminum sulfate in DPBS, AH in 5 M sodium acetate buffer, or AH in PBS), with varying amounts of aluminum (i.e., 30, 60, or 90 μg Al), in a total volume of 50 μL. Mice were immunized at weeks 0, 3, and 6. At week 10, half of each group was euthanized, and relevant tissues were harvested from these mice for further analysis. Mice were bled prior to each immunization and at 2-week intervals for 20 or 23 weeks. Blood samples were centrifuged at 14,000× *g* for 5 min at 4 °C. Serum was collected and stored at −25 °C until use.

### 2.7. IgG and IgG2a ELISAs

The assay was performed as previously described [16]. Briefly, 96-well Immulon^®^ high-binding U-bottom plates were coated with 100 μL of either HIV-1 A244 gp120 protein (1 µg/mL) or CRM_197_ (1 µg/mL) in DPBS (pH 7.4) and stored overnight at 4 °C. The plates were then blocked with 250 μL of blocking buffer, PBS containing 0.5% milk, and 0.1% Tween^®^ 20 at room temperature (RT) for 2 h. Individual serum samples were diluted in blocking buffer to an appropriate starting dilution and added to the first row of the 96-well plates in triplicates, where they were serially diluted two-fold in the blocking buffer in each row of the plates. The plates were then incubated at RT for 1 h, followed by the addition of horseradish peroxidase-conjugated goat anti-mouse secondary IgG (1:40,000 dilution in blocking buffer) and incubation at RT for 1 h. Color was developed by addition of peroxidase substrate components A and B and incubation in reduced lighting at RT for 1 h. The reaction was stopped by the addition of 100 μL per well of 1% sodium dodecyl sulfate, and the absorbance was measured within 30 min at 405 nm in a Spectramax M2 microplate reader (Molecular Devices, San Jose, CA, USA). The results are expressed as endpoint titers defined as the reciprocal dilution that gives an absorbance value that is greater than, or equal to, twice the background value (in an antigen-coated well that did not contain serum but had all the other components added). Antibody-positive (anti-A244 gp120 or anti-CRM_197_ seropositive samples from other studies) and antibody-negative controls were included on each plate.

Subclass IgG2a ELISAs were performed in a similar manner as described above for the antigen-specific IgG ELISA, with few modifications. This was performed on NUNC Maxisorp^TM^ 96-well flat-bottom plates. An HRP-conjugated goat anti-mouse IgG2a secondary IgG (1:1000 dilution in blocking buffer) was used. Mouse IgG2a standard was utilized as a positive control.

### 2.8. Statistical Analyses

Statistical comparison between multiple groups was performed using an ordinary one-way ANOVA with Tukey’s multiple-comparison test. Statistical comparison between 2 groups was performed using unpaired *t*-test with Welch’s correction. Differences among values are statistically significant if *p* ≤ 0.05.

## 3. Results

### 3.1. Solubility of Aluminum Chloride in PBS and Sodium Acetate Buffers

The solubilities of the individual test materials were readily characterized by visual inspection, using Alhydrogel^®^ as a positive control. As shown in Figure 2, 1.5 mg Al/mL aluminum chloride in 5 M sodium acetate buffer remained visually soluble as a function of time at various ranges of different storage temperatures. Aluminum chloride in sodium acetate maintained its solubility for 4 h at room temperature (RT). At 4 °C, there were no visible particulates in the solution for >10 weeks (shown in Figure 1 are photographs up to 48 h). Further, we observed that freezing the solution for 24 h at −20 °C did not result in the formation of particulates during re-solubilization, suggesting that freezing had no effect on the solubility of aluminum chloride in 5 M sodium acetate.

**Figure 3:**
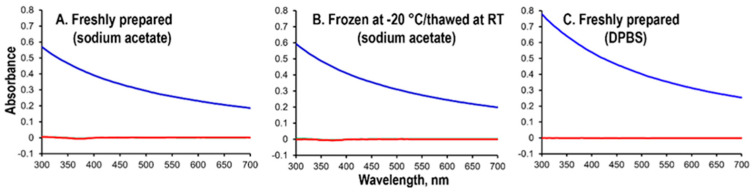
UV–visible spectra of particulate Alhydrogel suspension and soluble aluminum chloride and aluminum triacetate. (**A**) Absorbance scans of freshly prepared AH (blue), aluminum chloride and aluminum triacetate (red, shown in two overlapping traces) in sodium acetate; (**B**) absorbance scans of frozen/thawed AH (blue), aluminum chloride and aluminum triacetate (red, shown in two overlapping traces) in 5 M sodium acetate; and (**C**) absorbance scans of freshly prepared AH (blue), aluminum chloride and aluminum triacetate (red, shown in two overlapping traces) in DPBS.

The solubility of aluminum chloride or aluminum triacetate in sodium acetate buffer was further characterized using UV–visible scattering experiments. As shown in Figure 3, Alhydrogel in sodium acetate exhibited absorbance/scattering from 300 nm to 700 nm, while there was no absorbance/scattering observed in the freshly prepared and freeze-thawed aluminum chloride and aluminum triacetate (Figure 3A,B). Since aluminum phosphate in aqueous systems is particulate in nature at pH ~3.0 to ~7.5 [17], this observation further confirmed the solubility and the absence of particulate aluminum chloride and aluminum triacetate in sodium acetate buffer. A similar observation was found for aluminum chloride in DPBS (Figure 3C), where there was no absorbance/scattering observed from 300 nm to 700 nm, suggesting the absence of particulate aluminum phosphate salt in DPBS.

### 3.2. Immune Responses Induced with Aluminum Chloride and Aluminum Sulfate

#### Adjuvants in PBS

**Figure 4:**
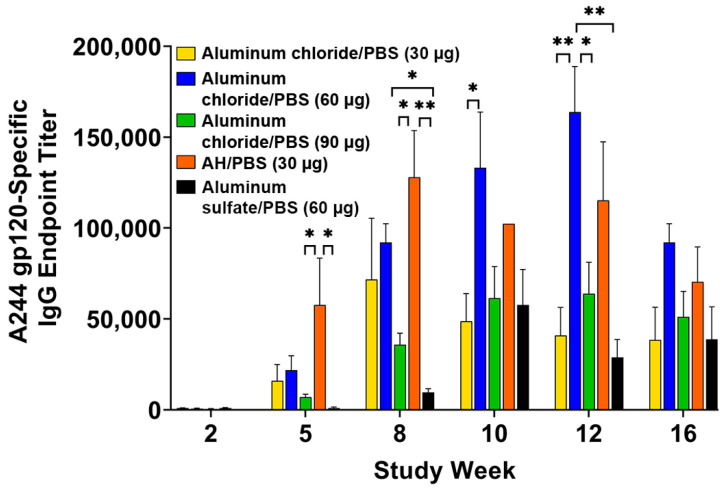
A244 gp120-specific IgG antibody responses in mouse serum induced with different concentrations of aluminum chloride/PBS (30, 60, 90 µg Al), AH/PBS (30 µg Al), and aluminum sulfate/PBS (60 µg Al). Each bar represents the mean ± SEM of individual serum samples (n = 5), analyzed in triplicate. * *p* < 0.05; ** *p* < 0.01.

The adjuvant activities of aluminum chloride (30, 60, and 90 μg Al), and aluminum sulfate (60 μg Al), in comparison to AH (30 μg Al) in PBS, with A244 gp120 as an antigen, were investigated in mice. As shown in Figure 4, immunization with A244 gp120 adjuvanted with aluminum chloride, aluminum sulfate, or AH resulted in variable induction of immune responses to A244 gp120. Formulation with 60 μg of Al in aluminum chloride mounted higher anti-A244 gp120 endpoint titers compared to those with 30 μg or 90 μg of Al, particularly at weeks 10 and 12. Starting at week 8 (2 weeks after the third immunization), anti-A244 gp120 endpoint titers elicited with both aluminum chloride/PBS and AH/PBS adjuvants were essentially equivalent. In contrast, aluminum sulfate adjuvant was consistently inferior at weeks 5–12, with endpoint titers significantly lower compared to those of aluminum chloride (60 μg Al) and AH (30 μg Al) (Figure 4).

**Figure 5:**
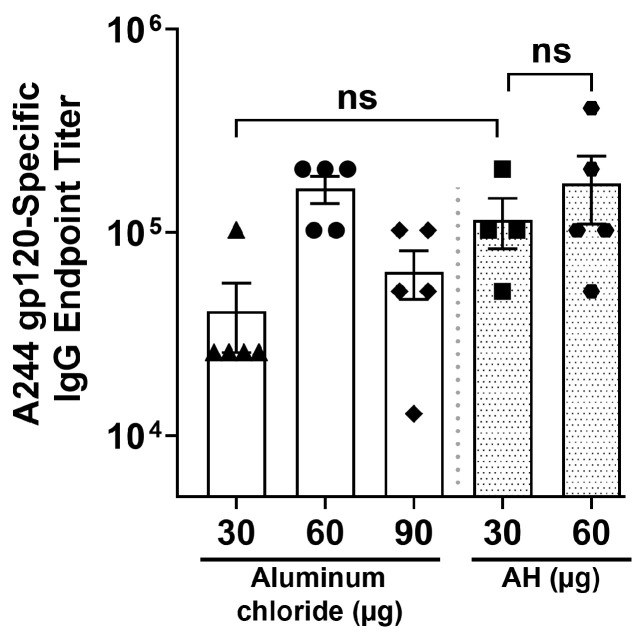
A244 gp120-specific IgG antibody titers at week 12 in mouse serum induced with different concentrations of aluminum chloride/PBS (30, 60, 90 µg Al), AH/PBS (30 µg Al), and AH/sodium acetate (60 µg Al). Each bar represents the mean ± SEM of individual serum samples (n = 5), analyzed in triplicate. ns: not significant.

As depicted in Figure 5, at week 12, the anti-A244 gp120 endpoint titers induced with 30 μg of Al in AH/PBS and 60 μg of Al in AH/sodium acetate were statistically equivalent. The endpoint titers elicited by AH in PBS or sodium acetate were equivalent to those induced with aluminum chloride (60 μg Al) adjuvant.

### 3.3. Immune Responses Induced with Aluminum Chloride and AH in Sodium

#### Acetate Buffer

**Figure 6:**
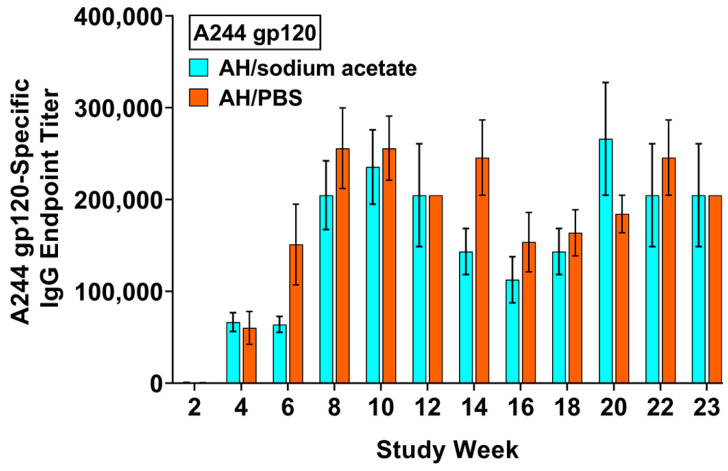
A244 gp120-specific IgG antibody responses in mouse serum induced with AH/sodium acetate (30 µg Al) and AH/PBS (30 µg Al). Each bar represents the mean ± SEM of individual serum samples (n = 10 for AH/sodium acetate through week 10; n = 5 for all others), analyzed in triplicate.

In water, aluminum chloride (40 mg/mL) is strongly acidic, with a pH of ~2.9. From a safety standpoint, aluminum chloride in water (pH ~2.9) or PBS (pH ~3.5) is not practical for use as a vaccine adjuvant. Thus, we explored different buffer systems that can adjust the pH to ~7.0, while maintaining their solubilities. As shown in Figure 1, the acidity of aluminum chloride was neutralized by 5 M sodium acetate, raising the pH from ~2.9 to ~7.0, without any visual precipitation of aluminum salts. In the subsequent experiments using adjuvant formulations in sodium acetate, a pH range of 6.0–7.0 was maintained. As shown in Figure 6, the anti-A244 gp120 endpoint titers induced with AH in sodium acetate were statistically similar to those in PBS throughout the duration of the study.

**Figure 7:**
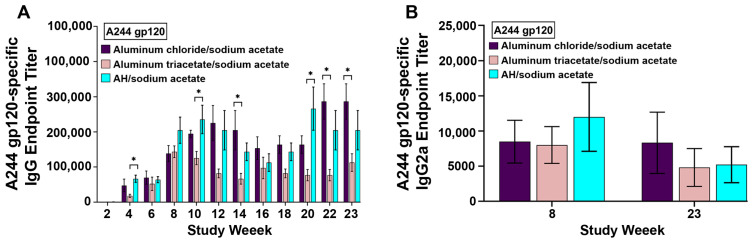
(**A**) A244 gp120-specific IgG antibody responses in mouse serum with aluminum chloride/sodium acetate (60 µg Al), aluminum triacetate/sodium acetate (60 µg Al), and AH/sodium acetate (30 µg Al). (**B**) A244 gp120-specific IgG2a antibody responses in mouse serum with aluminum chloride/sodium acetate (60 µg Al), aluminum triacetate/sodium acetate (60 µg Al), and AH/sodium acetate (30 µg Al). Each bar represents the mean ± SEM of individual serum samples (n = 10 through week 10; n = 5 for weeks 12–23), analyzed in triplicate. * *p* < 0.05.

In sodium acetate buffer, aluminum chloride can potentially react with acetate ions, forming aluminum triacetate via a chloride ligand exchange reaction [18]. Because of this, we investigated the utility of aluminum triacetate as an adjuvant in comparison with aluminum chloride and AH in sodium acetate buffer. Similarly, aluminum triacetate was also soluble in sodium acetate buffer at pH ~6.5. As shown in Figure 7A, the antibody endpoint titers to A244 gp120 induced with aluminum triacetate were statistically similar to those of aluminum chloride, except for weeks 14, 22, and 23, and were also statistically comparable to those of AH, except for weeks 4, 10, and 20. The formulations adjuvanted with soluble aluminum chloride and aluminum acetate exhibited IgG2a endpoint titers that were not statistically different than those obtained with particulate Alhydrogel (Figure 7B).

**Figure 8:**
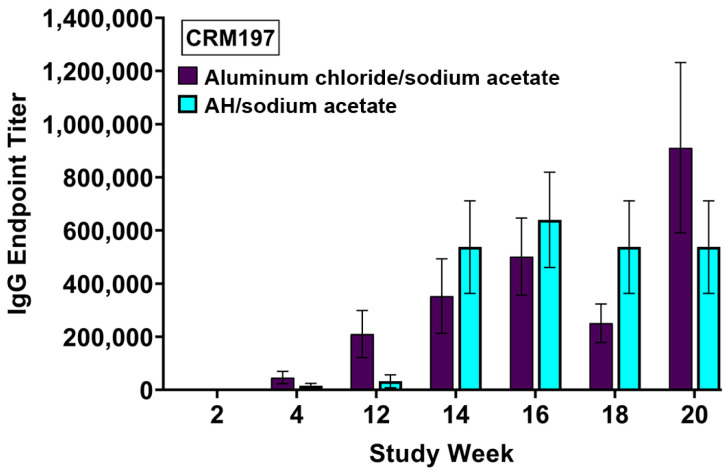
CRM_197_-specific IgG antibody responses in mouse serum induced with aluminum chloride/sodium acetate (30 µg Al) and AH/sodium acetate (60 µg Al). Each bar represents the mean ± SEM of individual serum samples (n = 5).

The adjuvant activity of aluminum chloride in sodium acetate was also tested with CRM_197_ as an antigen in mice. As shown in Figure 8, immunization with CRM_197_ adjuvanted with aluminum chloride in sodium acetate resulted in the induction of anti-CRM_197_ antibodies with endpoint titers that are statistically equivalent to those of AH.

## 4. Discussion

As indicated in the Introduction, the major goal of this work was to determine whether a water-soluble aluminum salt that has adjuvant activity equivalent to an established particulate aluminum salt could be identified. Here, we have discovered three water-soluble aluminum salts that exhibited adjuvant activity: aluminum chloride, aluminum sulfate, and aluminum triacetate. We have demonstrated that aluminum chloride buffered with sodium acetate remained soluble after being frozen at −20 °C and thawed in a water bath at room temperature (22 °C). Under the conditions used, soluble aluminum chloride buffered by sodium acetate produced an adjuvanticity comparable to that of a widely used commercial particulate aluminum hydroxide adjuvant (Alhydrogel^®^) when tested with two proteins as model antigens: HIV-1 A244 gp120 or CRM_197_. Alhydrogel^®^ was chosen for comparison because it is a widely used commercial source for research studies; however, other commercial adjuvants could have been similarly used [19,20]. In addition, a previous investigation showed that the freezing and thawing of Alhydrogel^®^ resulted in significant structural and chemical damage [13]. Because aluminum chloride buffered with sodium acetate exhibited an adjuvanticity equivalent to Alhydrogel^®^ within a pH range of 6.0–7.0, and because it is freezable, it might be considered a candidate soluble aluminum salt that could substitute for particulate aluminum salts as a vaccine adjuvant.

As noted in the Introduction, particulate aluminum salts have certain manufacturing vulnerabilities, of which the inability to sterile filter the final product is one. However, after manufacture of the adjuvanted vaccine, the requirement to maintain the temperature between 2 °C and 8 °C without even a single period of freezing during the entire worldwide cold-chain transportation and storage process before local administration of the vaccine to the individual has been recognized as a major vulnerability. Simple understanding that in the cold chain vaccines might be stored in “…walk-in coolers, domestic refrigerators, ice-lined refrigerators, kerosene electric refrigerators, purpose-built refrigerators, bar-type refrigerators, cold boxes, vaccine carriers and study boxes” [15] gives some idea of the enormous complexity during transportation and storage that could be at risk with accidental malfunction.

Particulate aluminum salt adjuvants are often cited as being inexpensive because aluminum is the third most common element on earth, and particulate aluminum salts are commonly used in anti-perspirants, antacids, cosmetics, and toothpaste [21,22]. While a low cost for obtaining mined aluminum salt may be true for the manufacture of aluminum salt adjuvants, the increased cost during the cold chain that is required to prevent modern manufactured adjuvanted vaccines from freeze-damage is rarely considered. This is a cost that also includes the possibility of enhanced vulnerability to disease due to the lack of protection of the vaccinated individual when the vaccine has been inadvertently freeze-inactivated [15].

Starting with adjuvant system AS04, which contains monophosphoryl lipid A adsorbed to particulate aluminum salt, which is now used both in a hepatitis B vaccine and in a vaccine against several types of cancer caused by human papilloma virus, and also with CpG adsorbed to aluminum salt in a hepatitis B vaccine, there has emerged considerable interest in vaccines that adsorb additional adjuvants to aluminum salt [23,24]. These more expensive vaccines that contain adjuvant molecules adsorbed to particulate salt emphasize that the accidental freezing of the particulate aluminum salt at any point in the extended cold chain further increases the potential costs associated with these new vaccines.

Many previous studies have generated numerous complicated theories regarding the mechanisms by which particulate aluminum salts exert their adjuvant activities, but there is still no definitive understanding of the adjuvant mechanisms [7,23,25,26,27,28,29,30]. Presumably, based on the data in our study, the long-held idea originally offered by Glenny et al. [31], and reiterated by others, that the clumped particles of aluminum salt containing antigen serve as a depot for prolonged release of the antigen might be further eliminated here as a primary aluminum salt adjuvant mechanism. The availability of a soluble aluminum salt adjuvant as described here might be useful for future investigations for sorting through some of the previous complexities of theories of aluminum adjuvant mechanisms.

As described above, the mechanism of freezing, leading to reduced immunogenicity of vaccines containing aluminum salt adjuvants, is strongly and convincingly attributed to freeze-induced damage to the aluminum salt particles themselves. In view of this, soluble aluminum salt adjuvants lacking any particles should eliminate any damage caused by freezing, and this presumably would allow the differentiation between freeze-damage to the aluminum salt adjuvant and freeze-damage, if any, to the antigen itself. Current studies are underway to determine whether sequential, or multiple, or different durations of freezing of a soluble aluminum salt has any adverse effect on the adjuvanticity.

## 5. Conclusions

Soluble aluminum salts, such as aluminum chloride, aluminum sulfate, or aluminum acetate (also known as aluminum triacetate) exhibit enhanced immunity as adjuvants for induction of murine antibodies that are equivalent to those exhibited by particulate commercial aluminum hydroxide (Alhydrogel^®^). If needed, buffering to a suitable pH can be achieved with sodium acetate, while maintaining the solubility of the adjuvant.

## Data Availability

All data are available from the authors.
